# A New Mediterranean Lifestyle Pyramid for Children and Youth: A Critical Lifestyle Tool for Preventing Obesity and Associated Cardiometabolic Diseases in a Sustainable Context

**DOI:** 10.1016/j.advnut.2025.100381

**Published:** 2025-01-21

**Authors:** Rosa Casas, Ana María Ruiz-León, Jesús Argente, Cesarettin Alasalvar, Aadil Bajoub, Isabel Bertomeu, Margherita Caroli, Sara Castro-Barquero, Fatima Crispi, Jacques Delarue, Rodrigo Fernández-Jiménez, Valentin Fuster, Javier Fontecha, Paz Gómez-Fernández, Jordi González-Juste, Christina Kanaka-Gantenbein, Eirini Kostopoulou, Rosa M Lamuela-Raventós, Yannis Manios, Ascensión Marcos, Luis A Moreno, Sonia de Pascual-Teresa, Blanca Raidó-Quintana, Marta G Rivera-Ferre, Gloria Santos-Beneit, Iris Shai, Bessie E Spiliotis, Antonia Trichopoulou, Andrea Vania, Gregorio Varela-Moreiras, Anna Vila-Marti, Walter Willett, Emilio Ros, Ramon Estruch

**Affiliations:** 1 Centro de Investigación Biomédica en Red Fisiopatología de la Obesidad y la Nutrición (CIBEROBN), Instituto de Salud Carlos III (ISCIII), Madrid, Spain; 2 Department of Internal Medicine, Institut d’Investigacions Biomèdiques August Pi Sunyer (IDIBAPS), Hospital Clinic, University of Barcelona, Barcelona, Spain; 3 Institut de Recerca en Nutrició i Seguretat Alimentaria (INSA-UB), University of Barcelona, Barcelona, Spain; 4 Fundación Dieta Mediterránea, Barcelona, Spain; 5 Department of Pediatrics and Pediatric Endocrinology, Hospital Infantil Universitario Niño Jesús, Instituto de Investigación La Princesa, Department of Pediatrics, Universidad Autónoma de Madrid, Madrid, Spain; 6 Research Program on Childhood Obesity, IMDEA Food Institute, Madrid, Spain; 7 Life Sciences, TÜBİTAK Marmara Research Center, Gebze-Kocaeli, Turkey; 8 Laboratory of Food and Food By-Products Chemistry and Processing Technology, Ecole Nationale d’Agriculture de Meknès (ENAM), Meknes, Morocco; 9 Independent Researcher, Francavilla Fontana, Brindisi, Italy; 10 Department of Nutrition, Harvard T.H.Chan School of Public Health, Harvard University, Boston, MA, United States; 11 BCNatal Fetal Medicine Research Center (Hospital Clínic and Hospital Sant Joan de Déu), IDIBAPS, Barcelona, University of Barcelona, Spain; 12 Centre for Biomedical Research on Rare Diseases (CIBER-ER), ISCIII, Madrid, Spain; 13 Department of Nutritional Sciences and ER7479 SPURBO, University Hospital/Faculty of Medicine/University of Brest, France; 14 Hospital Universitario Clínico San Carlos, IdiSSC, Madrid, Spain; 15 Centro de Investigación Biomédica En Red en enfermedades CardioVasculares (CIBERCV), ISCIII, Madrid, Spain; 16 Centro Nacional de Investigaciones Cardiovasculares (CNIC), Madrid, Spain; 17 Foundation for Science, Health and Education (SHE), Barcelona, Spain; 18 The Zena and Michael A. Wiener Cardiovascular Institute, Icahn School of Medicine at Mount Sinai, New York, NY, United States; 19 Department of Bioactivity and Food Analysis, Food Lipid Biomarkers and Health Group, Institute of Food Science Research, CIAL (CSIC-UAM), Universidad Autónoma de Madrid, Madrid, Spain; 20 Department of Anthropology, Universidad Nacional de Educación a Distancia (UNED), Madrid, Spain; 21 Division of Endocrinology, Diabetes and Metabolism, First Department of Pediatrics Medical School, National and Kapodistrian University of Athens, Aghia Sophia Children’s Hospital, Athens, Greece; 22 Division of Paediatric Endocrinology and Diabetes, Department of Paediatrics, University of Patras School of Medicine, Patras, Greece; 23 Departament de Nutrició, Ciències de l’Alimentació i Gastronomía, Facultat de Farmacia i Ciències de l’Alimentació, University of Barcelona, Barcelona, Spain; 24 Department of Nutrition and Dietetics; Harokopio University, Athens, Greece; 25 Department of Metabolism and Nutrition, Institute of Food Science, Technology and Nutrition (ICTAN), Spanish National Research Council (CSIC), Madrid, Spain; 26 Growth, Exercise, Nutrition and Development (GENUD) Research Group, Instituto Agroalimentario de Aragón (IA2), University of Zaragoza, and Instituto de Investigación Sanitaria de Aragón (IIS Aragón), Zaragoza, Spain; 27 Department of Fundamental Care and Medical-Surgical Nursing, School of Nursing, Faculty of Medicine and Health Sciences, University of Barcelona, Barcelona, Spain; 28 INGENIO (CSIC-Universitat Politècnica de Valéncia), València, Spain; 29 Faculty of Health Sciences, Ben-Gurion University of the Negev, Beer-Sheva, Israel; 30 Department of Pediatrics, University of Patras School of Medicine, Patras, Greece; 31 Academy of Athens, Hellenic Health Foundation; Athens, Greece; 32 Independent Researcher, Rome, Italy; 33 Department of Pharmaceutical and Health Sciences, Faculty of Pharmacy, CEU San Pablo University, Madrid, Spain; 34 Research Group M30—Methodology, Methods, Models and Outcomes of Health and Social Sciences and Welfare, Universitat de Vic—Universitat Central de Catalunya, Vic, Spain; 35 IDIBAPS, University of Barcelona, Barcelona, Spain

**Keywords:** adolescents, children, Mediterranean diet, lifestyle, obesity, cardiometabolic diseases, extra-virgin olive oil, sustainability

## Abstract

Cardiovascular disease risk factors begin in childhood and track into adulthood, increasing the possibility of impaired cardiometabolic health. Adopting healthy dietary patterns can help curb childhood obesity, a worrisome epidemic problem at present. In the era of personalized nutrition, dietary recommendations should be adapted to different stages of life, including children (older than 3 y) and adolescents. In this study, we present an updated version of the Mediterranean Lifestyle Pyramid addressed to children and adolescents, which may be used as a prevention tool by health professionals, teachers, and stakeholders. This pyramid arises from a consensus position between participants in an International Congress on Mediterranean diet held in Barcelona. During this meeting, after reviewing all literature published, a consensus was reached on the new Mediterranean Lifestyle Pyramid for kids including details such as labels of the pyramid, position of foods, servings, type of foods, and healthy lifestyle habits. All components of the pyramid are supported by the most recent scientifically sound research and are based upon top-level evidence in nutritional sciences. Fruit, vegetables, legumes, nuts, wholegrains, and extra-virgin olive oil continue to be at the basis of the pyramid, but the importance of an adequate intake of fish, dairy products, and meat during these particular ages, when body and brain development occurs, is also considered. The promotion of physical activity, adequate sleep, and good emotional health are emphasized, as well as the consumption of seasonal and local products, and overall sustainability. Improving dietary habits in early stages of life should increase health in adulthood and reduce future incidence of noncommunicable chronic diseases. The Mediterranean diet and its graphic representation in the Lifestyle Pyramid should be a health-fostering tool not only for adults and children but also for the entire planet because it promotes the diversity of species, respect for the earth, and the local economy.

**Statements of Significance**

A new Mediterranean Lifestyle Pyramid is addressed to children and adolescents, as well as health professionals, teachers, and stakeholders. Fruit, vegetables, legumes, nuts, wholegrain cereals, and extra-virgin olive oil continue to be a crucial part of the pyramid, but the importance of adequate intake of fish, dairy products, and meat in these early ages, when body and brain development occurs, is also emphasized.

## Introduction

Cardiovascular disease (CVD) risk factors present in children and adolescents track into adulthood, wherein they are predictive of impaired cardiometabolic health and higher rates of CVD. Over the last 3 decades, there has been a sustained epidemic of childhood and adolescent obesity. In the United States, the percentage of youth aged 2–19 y with obesity reached 19.7% in the period 2017–2020 [1], whereas in the WHO European Region, overweight and obesity affect nearly 1 in 3 children (29% of boys and 27% of girls) [2], thus making excessive body weight by far the most prevalent CVD risk factor in childhood. Low-income and middle-income countries are not spared this epidemic, while also having to cope with undernutrition [3].

Overweight and obesity have a multifactorial etiology, but unhealthy dietary habits, along with physical inactivity, are among the most important factors for the development of obesity-associated cardiometabolic complications such as CVD and other noncommunicable diseases (NCDs). Excessive body weight is important at younger ages because it predicts adverse health outcomes throughout life, including heart disease, hypertension, dyslipidemia, diabetes, stroke, certain cancers, and osteoarthritis. Comorbidities of obesity, such as type 2 diabetes mellitus (T2DM), metabolic dysfunction–associated steatotic liver disease, and depression are not infrequent in adolescents with obesity. Moreover, obesity in children is strongly associated with low self-esteem and a negative body image [4].

Research has shown that diet and exercise are the mainstay of obesity treatment [5]. However, whether diet or exercise separately or combined is more effective at weight loss in children and adolescents with overweight or obesity remains unclear [6,7]. Studies in adults have reported that higher adherence to the Mediterranean diet (MeDiet), the traditional, plant-based dietary pattern of countries in the Mediterranean basin, is inversely related to overweight and obesity [5], whereas data in children suggest that compliance with the MeDiet is associated with lower body weight independently of physical activity [[8], [9], [10], [11]]. Accumulated evidence indicates that high adherence to the MeDiet is linked to lower all-cause mortality and decreased incidence of CVD, metabolic syndrome, T2DM, all-cause dementia, and Alzheimer disease and other neurodegenerative diseases, and cancer [[12], [13], [14], [15], [16], [17], [18], [19]].

Currently, the MeDiet, which was customary in Mediterranean countries, is reputed as one of the healthiest dietary patterns worldwide. This dietary model is based on the traditional diets in Crete (Greece) and southern of Italy in the early1960s [20]. A large prospective study in European children suggests that compliance with the MeDiet is associated with lower body weight independently of age, sex, socioeconomic status, and physical activity [11]. Noticeably, in that study, children from Mediterranean countries had lower adherence to the MeDiet than those from Nordic countries. A review of dietary surveys in adolescents that includes 24 reports from Mediterranean countries also underlines the medium-low adherence to the MeDiet in this population, which is associated with the rise in overweight and obesity in this region [21]. The findings of randomized controlled trials of nonenergy-restricted MeDiet interventions compared with control diets in children and adolescents, mostly conducted in Mediterranean countries, support a beneficial effect of the MeDiet against overweight/obesity in this population [22].

The MeDiet is characterized by the use of extra-virgin olive oil (EVOO) as the main source of fat, high consumption of fruits and vegetables, whole grains, legumes, seeds, nuts, fish, and shellfish; moderate consumption of eggs, dairy products like cheese and yogurt, lean meat (poultry), and moderate intake of red wine in adults [23]. However, due to the known adverse health effects of alcohol at an early age, children should not drink wine or any other alcoholic beverage. The MeDiet is more than a dietary pattern, as it involves a particular lifestyle which encompasses, among other aspects, slow eating and enjoying conversation around the table. Sharing cooking and meals are perfect social circumstances to exchange ideas and communicate with family members, friends, and neighbors and to forge closer ties in cordiality and respect [24].

It is important to consider that the MeDiet has emerged from the synergy between the different traditions present on both sides of the Mediterranean basin, a product of the exchange of foods and cooking techniques since ancient times. In addition, the arrival of American foods such as potatoes, tomatoes, pepper, and beans (a whole gastronomical and nutritional revolution on its own) to Mediterranean countries turned foods that were mere “accompaniments” of local products into main dishes and protagonists of Mediterranean cuisine and dining: a mix of traditions concerning agriculture, fishing and livestock, food processing, preservation, cooking techniques, food sharing, and consumption [24]. Additionally, the MeDiet plays a large role in culture, as it is present in festivities, celebrations, and daily life, represented in crafts, in markets, as places for exchange, or within the familiar nucleus, where techniques and recipes are transmitted to the next generations [24].

Apart from being recognized as a healthy dietary pattern, the MeDiet is also considered a sustainable diet with a low environmental impact. The FAO of the United Nations highlights its role in protecting and respecting biodiversity and ecosystems, suggesting that it is an accessible, economically fair and affordable, nutritionally adequate, safe, and healthy diet, which is accomplished while optimizing natural and human resources [[25], [26], [27]]. Many studies have now confirmed this fact. When comparing the MeDiet with other dietary patterns, as well as with current consumption trends in some Mediterranean countries, the MeDiet scores better for several indicators, including greenhouse gas emissions, water usage, and ecologic and carbon footprint [28,29], particularly due to the low-to-moderate consumption of red meat within this dietary pattern.

However, the Mediterranean diet must extend beyond a simple eating pattern. In Greek, the word “diatia,” from which the word “diet” is derived, means a holistic way of living, or lifestyle, which includes being active and productive, engaging in daily moderate exercise, managing overall stress, sleeping well, and maintaining close relationships with family and friends. Thus, when discussing exercise and/or sleep besides diet, a suitable name for the new pyramid would be Mediterranean Lifestyle Pyramid (MedLifestyle).

In addition, proposals have been made as well to update the MedLifestyle pyramid to include environmental aspects [29,30]. It goes without saying that transporting foods over long distances, along with packaging and preserving food products, requires large amounts of energy and resources and contributes to greenhouse gas emissions.

Changes in the food environment linked to globalization have resulted in more processed and energy-dense foods being increasingly available, accessible, and affordable, which, together with aggressive marketing directed to children of so-called ultraprocessed foods (UPFs), are likely major drivers of both the progressive abandonment of the MeDiet and excess energy intake and weight gain among different populations, children being particularly vulnerable [4]. Factors other than pervasive consumption of UPFs endanger the legacy of the MeDiet for future generations: tourism and migration, changes in intergenerational relationships, sedentary lifestyles (social networks, videogames, and over-the-top platforms), desocialization, loss of traditional habits, changes in chronobiologic rhythms of food intake, and little time to cook, which prompts abandoning fresh, natural foods and substituting them for ready-to-eat prepared products [30].

In recent years, young people have increased their consumption of foods that are high in energy but low in nutritional density (e.g., soft drinks, pastries, cakes, and sweets) or high in sugars and SFAs (e.g., meat, meat products, dairy products, and salty prepackaged snacks). Youth tend to have low fiber intake (e.g., reduced amounts of whole grains and legumes) and a reciprocal increase of refined carbohydrates, with reduced intakes of antioxidants and vitamins due to decreased consumption of fruits and vegetables. This reflects a decline in the Mediterranean diet among the young [31,32].

Young people are easily influenced by media, fashion trends, food environment, and food advertisements and, at present, they acquire their tastes and preferences mainly outside the family, being more attracted to simplified and industrialized foods than to those included in the traditional MeDiet. The cross-sectional HELENA (Healthy Lifestyle in Europe by Nutrition in Adolescence) study reported that, in 3528 (52% females) urban European adolescents aged 12 to 17 y, the consumption of fruits and vegetables as well as milk and dairy products was lower than recommended. On the contrary, the consumption of meat and meat products, fat-rich foods, and sweets was higher than recommended [32]. In relation to beverage intake, the HELENA investigators reported that most adolescents drank water, followed by sugar-sweetened beverages (SSBs), sweetened milk, low-fat milk, and fruit juice, which provided the largest amount of energy [32]. Additionally, intakes of SFAs and salt were higher than recommended, whereas PUFA intake was lower.

To preserve and disseminate the cultural heritage of the MedLifestyle to future generations and improve salutary eating habits, nutritional programs directed to children and adolescents are warranted. Healthy lifestyles should be encouraged at every life stage, but childhood and adolescence are key time periods to promote and establish them throughout the lifecourse.

## Methods

For this narrative review, we conducted a comprehensive search of MEDLINE and EMBASE through September 2024 for English language reports of epidemiologic and clinical studies and meta-analyses thereof describing the effects of the Mediterranean diet and its key components on cardiometabolic outcomes, particularly those related to body weight, in children and adolescents. We also searched for studies reporting compliance with food recommendations in general and the Mediterranean diet in particular among young persons. Finally, we searched references cited in original studies and reviews identified, together with articles citing landmark clinical studies, reviews, and meta-analyses, as provided by the publishers of individual articles in their websites. Data were examined for relevance, quality, and consistency and independently extracted by the authors, who reached a consensus when in doubt about a specific citation.

Since the 1990s, the MeDiet guideline for adults has been represented as a pyramid, wherein the frequency of consumption and serving sizes are highlighted graphically [23,24,29]. We acknowledge that the representation of the Mediterranean lifestyle with a pyramid has been an excellent initiative to promote its knowledge and adoption, which is difficult to surpass. The first food pyramid reflecting Mediterranean traditions was depicted in a 1995 article coauthored by investigators from the Harvard School of Public Health and Greek nutrition experts [23]; the second Mediterranean Lifestyle Pyramid article, published in 2011, was promoted by the Spanish Mediterranean diet Foundation with nutrition investigators from various Mediterranean countries [24]. Both the 1995 and 2011 Mediterranean Lifestyle Pyramid articles were directed to adults. However, development and maintenance of good health during childhood is a top public health priority.

In addition, indexes of adherence to the MeDiet have been increasingly used to assess its salutary effects and preventive potential against development of NCDs [31]. A contemporary MeDiet pyramid should consider all scientific evidence for the health benefits of this dietary pattern, reflecting not only traditional foods (e.g., grains, fruits, vegetables, pulses, nuts, or olive oil) but also potatoes, tomatoes, peppers, or beans that have been brought in from America after the 16th century and now are an integral part of the MeDiet. It is imperative that the present pyramid considers concepts such as seasonality, fresh and locally grown products, biodiversity, traditional and local products, and sustainability, as well as physical activity, the sleep pattern, and socializing. Culinary techniques should also be considered. For vegetables, boiling with little water is regarded as one of the healthiest cooking methods because it retains most of the nutrients in food. Slowly stewing meat or fish with vegetables, legumes, or potatoes preserves the nutrient components of the complex matrices of these foods, including bioactive nutrients and phytochemicals. Tomato and onion olive oil–based sauces are ideal to enhance the nutritional value and tastiness of rice and pasta. Either shallow pan frying or deep frying with EVOO are traditional cooking Mediterranean techniques that, if used correctly, much enhance the fried food nutritional quality and tastiness [33].

Currently, when reflecting on the diet of children, we must keep in mind that they do not have the ability to acquire, transform, or cook food by themselves, but they do influence the organization of the family diet with their tastes. In traditional life, the children’s daily diet was very soon the same as that of grown-ups, perhaps somewhat less spicy, but basically the whole family ate the same meals at the same time, without differences among family members; neither were there differences in the meals for festive days; except that perhaps children would be offered more sweets, but this was not usual. There was no specific diet for children; from 2 y of age, they ate the same meals as adults with a similar daily organization and hourly distribution of food, thus acquiring good dietary habits since an early age.

In order to achieve an updated version of the MedLifestyle pyramid for children and adolescents, a meeting was organized with members of the Mediterranean diet Foundation and a panel of international experts in Barcelona in April 2018. An extensive review of the available literature on this issue was distributed to the members. After several deliberations and meetings, a consensus was reached on a new MedLifestyle pyramid for kids. This includes details such as pyramid labels, food positions, servings, types of food, culinary techniques, and healthy lifestyle habits. All components of the pyramid are supported by the most recent scientifically sound research and are based upon top-level evidence in nutritional sciences. To this end, recent large epidemiologic studies, clinical trials, and meta-analysis thereof have been reviewed to extract the conclusions for the present scientific consensus.

## Results and Discussion

### Nutrition requirements

In addition to highlighting the imbalanced diet of a large sample of European adolescents, the previously mentioned HELENA study provided interesting data regarding nutritional intakes. In that survey, the main food groups contributing to energy intake were fat-rich foods, solid sweets, and SSBs, followed by dairy foods and meats. Insufficient vitamin D intake was observed in practically all individuals, whereas vitamin E, folate, calcium, magnesium, and iodine appeared to be compromised, especially in girls [32].

Taking advantage of the Nutrition Facts label found on packaged foods and beverages, the United States FDA recommends monitoring the nutrient intake and obtain more dietary fiber, vitamin D, calcium, iron, and potassium, while reducing the intake of added sugars, sodium, and SFA [34]. Likewise, the Institute of Medicine and the Nutritional Objectives of the European Food Safety Authority [35] recommended that children and adolescents reduce fat intake, particularly SFA, and increase carbohydrate intake in all age groups. In addition, children should also decrease protein intake, mainly animal protein because many reach and surpass the upper limit of the Acceptable Macronutrient Distribution Range [11].

### Update of the Mediterranean Lifestyle Pyramid for kids (3–18 y)

**FIGURE 1:**
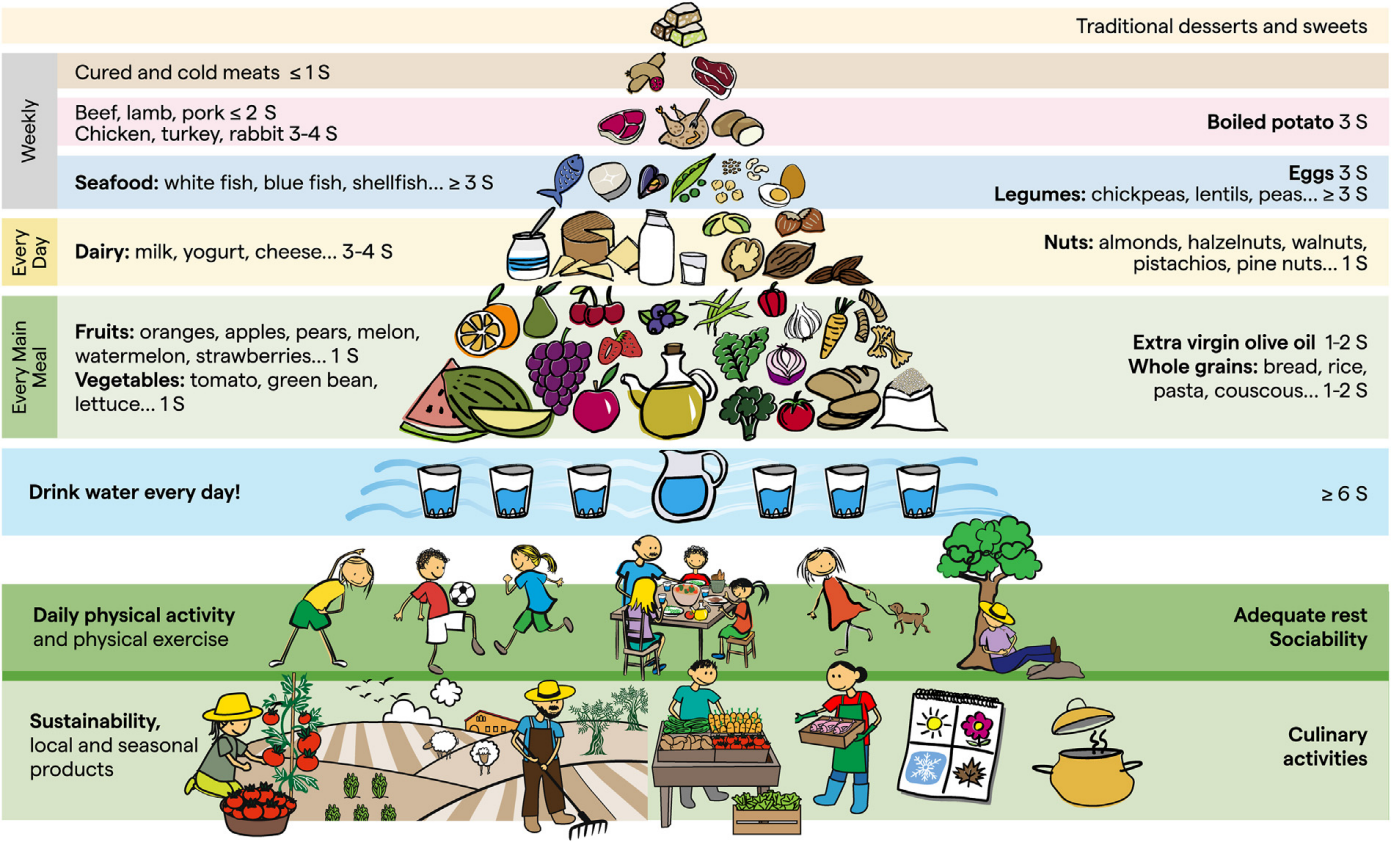
Mediterranean lifestyle pyramid for children and youth. A new Mediterranean Lifestyle Pyramid has been developed based on the healthy eating habits that should be followed by children and adolescents to achieve optimal health and mental development in these critical stages of life. The pyramid is based on sustainable products such as local and seasonal foods. Physical activity, conviviality, good emotional health, adequate sleep, and rest, as well as healthier culinary activities are promoted. Consumption every day of 5–8 glasses of water should be guaranteed. Extra-virgin olive oil, cereal products (preferably whole grain), fruits, and vegetables should be consumed in every meal; boiled potatoes may also be consumed ≤3 servings/wk, not exceeding 100 g/d; dairy products and nuts are recommended for daily consumption; seafood, eggs, pulses, and unprocessed white or red meat can be consumed weekly in different proportions. Cured meats and cold cuts, solid and liquid sweets, and traditional desserts should be consumed only occasionally. S, servings.

The food items depicted in the pyramid (Figure 1) have been selected considering that they are the most consumed in Mediterranean countries, the most representative according to seasonality, and those with the highest scientific evidence about their salutary effects. Thus, fruits such as melon, pears, strawberries, cherries, and blueberries have been added. In the group of vegetables, artichokes, green beans, carrots, peppers, tomatoes, broccoli, spinach, onions, and garlic have been included. The group of nuts has been expanded by depicting not only walnuts or hazelnuts but also pistachios and almonds. Regarding legumes, the consumption of chickpeas, beans, green peas, and lentils is highlighted. See also Supplemental Figure 1.

Graphically represented in the pyramid are also ancillary but important aspects of the Mediterranean lifestyle, including regular physical activity (individual or in-group); conviviality or sharing and enjoying conversation with family or friends at the table during a meal; biodiversity and seasonality; consumption of traditional, local, and ecofriendly products; and healthy culinary techniques. Table 1 presents a brief complementary text highlighting key points to follow the healthiest possible MeDiet.

**TABLE 1:** Decalogue of the Mediterranean diet.

1. Have cereals (preferably whole grain), fruits, and vegetables at every main meal. An important part of a healthy diet is vegetables and fruits. Not only is quantity important, one should also choose variety to cover nutritional needs because they are major sources of vitamins and minerals. They are also excellent sources of bioactives/phytochemicals, as well as fiber, and exhibit strong antioxidant activities. Their regular consumption can help prevent CVD and certain cancers. Since 100% fruit juice still contains free sugar, children should be encouraged to eat whole fruit instead. Additionally, they should consume vegetables to attain the desirable 5 fruit and vegetable servings per day recommendation. Potatoes in moderation (not french fries) count as vegetables (≤3 servings/wk). On the contrary, having cereals, preferably whole grain (bread, pasta, rice, and similar foods) on a daily basis provides an important part of the energy necessary for daily activities.
2. Use extra virgin olive oil as the main source of added fat for cooking and dressing dishes. It is rich in MUFA, vitamin E, and highly bioactive polyphenols with known cardioprotective properties. Limit use of salt and replace it by using herbs and spices to improve color and flavor in dishes.
3. Water is essential in the diet. Water is the perfect beverage, has no calories, and quenches thirst; it rehydrates the body and maintains its water balance. Drink between 1.5 and 2 L of water daily. Avoid sugary beverages because they provide empty calories and are associated with obesity and increased cardiometabolic risk.
4. Have dairy products (milk, yogurt, and cheese) and nuts every day. Dairy products are excellent sources of high biological value proteins, minerals (calcium, phosphorus, etc) and vitamins. A wide variety of nuts and seeds, without added salt or sugar, are also recommended to incorporate different nutrients for a healthy cardioprotective diet.
5. Choose seafood (fish and shellfish) and legumes as your main source of protein weekly. Three servings/wk of fish (2 of them oily fish) are recommended because its fats—although of animal origin—have properties very similar to fats from plants and are associated with reduced CVD rates. Moreover, consume ≥3 servings/wk of pulses, which improve cardiometabolic risk factors.
6. Eggs contain high-quality proteins, fats, and many vitamins and minerals that make them nutrient-rich foods. Consuming eggs in moderation (3 servings/wk) is a good alternative to meat and fish.
7. Limit consumption of red meat (maximum 2 servings/wk). Avoid processed meat (maximum 1 serving/wk). Replace red and processed meat by legumes, nuts, fish, poultry, or lean meat.
8. Fresh fruit should be the habitual dessert. Sweets and cakes can be consumed occasionally. Replace less healthy choices with more nutritious options like fermented dairy products (yoghurt and cheeses) or a handful of nuts.
9. Have an active daily routine and get enough sleep. Walk whenever possible, be active, and play sports. Try to do ≥60 min/d of moderate-intensity to vigorous-intensity physical activity, but the more the better! Getting good quality sleep is essential. Infants should sleep 10–13 h/d, schoolchildren around 10–12 h/d, and adolescents 8–10 h/d.
10. Share meals with family and friends and enjoy seasonal traditional dishes. Spend time with family and friends and participate in meal planning and preparation. Choose local, seasonal foods, and be as environmentally sustainable as you can!

Abbreviations: CVD, cardiovascular disease.

### Mediterranean dietary pattern

In addition to healthy culinary techniques, frequent consumption of the main food groups, serving sizes, and quality of foods should be considered to ensure an adequate and correct nutritional intake. The MeDiet is a plant-centered dietary pattern. To obtain a balanced diet, all main meals should include vegetables, fruits, cereals (preferably whole grain), and healthy fats such as EVOO. During the week, other sources of vegetable proteins, such as not only legumes but also dairy products, fish, meat, and eggs, must alternate in the main meals to obtain maximum positive effects on health. This dietary model should contribute to curtail total energy intake and help curb weight gain [[5], [28],^32^,^36^].

#### Each main meal

• One to 2 servings of wholegrains, such as bread, pasta, rice, bulgur, and couscous. Not only the quantity of carbohydrates but also their quality is important for health. Wholegrain cereals have a high amount of valuable nutrients such as fiber, vitamin E, B complex vitamins, iron, copper, zinc, magnesium, antioxidants, and bioactive phytochemicals compared with those that are refined [37,38]. Meta-analyses of prospective studies report that consumption of whole grains and their associated fiber is linked to lower all-cause and cause-specific mortality, particularly deaths from CVD, as well as incidence of CVDs, T2DM, and cancer [37,[39], [40], [41]]. In a meta-analysis of data from large cohort studies, glycemic index and load had associations of similar magnitude with cardiometabolic outcomes and total mortality, but in opposite directions as seen with cereal fiber [41]. Moreover, glycemic index was positively associated with weight change in a detailed analysis of 3 large cohorts [42]. These findings suggest that it is desirable to consume whole grains that are intact or coarsely milled, rather than finely milled, or pasta, which reduces glycemic index and does not contribute to weight gain within the context of a healthy diet [43].
• One serving of vegetables, including them raw in ≥1 meal/d to ensure optimal intake of vitamins and minerals. Their high nutrient density and content of dietary fiber, phytochemicals, and antioxidants, together with a low energy content, are beneficial to curtail adiposity and further overall health [44,45].
• One serving of fruits, especially whole fruits. Like vegetables, fruits have a high content of water, fiber, vitamins and minerals, and many nonnutritive salutary compounds, such as flavonoids, phenolic acids, carotenoids, and other antioxidants, which are associated with similar health benefits [45]. Although 100% fruit juice has a slightly lower fiber content and does not offer any nutritional advantage over the whole fruit, there has been controversy on its effects on body weight and cardiometabolic markers [46,47]. In general, 100% fruit or vegetable juice without added sugar does not promote adiposity or impair cardiometabolic risk factors [48], but it still contains free sugars. The WHO recommendations continue to emphasize limiting free sugars. Therefore, children should be encouraged to eat whole fruit instead of 100% fruit juice, incorporating ≥1 serving into every main meal.

Considering fruit and vegetables together, a recent meta-analysis concluded that, compared with the consumption of 2 servings/d of fruit and vegetables, consumption of 5 servings/d was associated with a 12% reduction of fatal CVD and a 13% decrease of all-cause mortality [49]. From a practical point of view, consuming 2 servings of vegetables and 3 of fruits is easier to achieve, especially among children, when 1 or 2 servings of fruit are made up of 100% fruit juice without added sugar [50].

#### Everyday

• Water: According to the European Food Safety Authority, an appropriate daily total water intake (which includes water from milk, other beverages, and foods) should be guaranteed. Requirements for boys and girls aged 2–3 y are 1300 mL/d, whereas for adolescents (14 y and older), requirements are 2 L/d for girls and 2.5 L/d for boys because they can be considered as adults in this regard [35]. Water is the perfect beverage; it has zero calories, quenches thirst, rehydrates the organism, and helps maintain the body water equilibrium.
• Dairy foods (yoghurt, cheese, and other fermented dairy products) should be consumed every day (3–4 servings/d), preferably as whole foods without added sugar or flavor. From childhood to adulthood, correct calcium intake is needed for strong and healthy bones [51]. Besides calcium-rich water, dairy foods not only are the main source of calcium but also contain other important nutrients such as protein, phosphorus, vitamin A, vitamin D (in whole milk or fortified skim dairy products), riboflavin, vitamin B-12, potassium, zinc, choline, magnesium, and selenium [51]. Other sources of calcium include green leafy vegetables, almonds, chickpeas, figs, fresh or canned small fish eaten with bones, like sardines and anchovies, and calcium-rich mineral water [52].
• EVOO should be present in every meal as the main source of dietary fat because of its high nutritional quality, which is characterized by a high content of MUFAs, vitamin E, bioactive polyphenols, and phytosterols [53]. Moreover, EVOO is more stable than other cooking oils against thermal injury and oxidation, giving rise to fewer harmful by-products during heating [33]. Thus, EVOO is recommended for frying and cooking. Importantly, EVOO has demonstrated vasculoprotective properties in clinical trials, whereas both EVOO and common olive oil have been associated with reduced CVD, T2DM, and all-cause mortality in prospective studies [[53], [54], [55]], and with reduced CVD rates within the context of a MeDiet in 2 seminal clinical trials: PREDIMED (Prevención con Dieta Mediterránea) [14] and CORDIOPREV (Coronary Diet intervention with olive oil and cardiovascular Prevention study) [56].
• Nuts should also be consumed daily (1–1.5 servings, equivalent to 30–45 g, per day). The term nuts encompasses tree nuts such as almonds, walnuts, pistachios, and hazelnuts, and peanuts, which are legumes albeit they have a similar nutrient profile and health effects as tree nuts. Nuts are excellent sources of salutary fats (MUFA and PUFA), protein, fiber, vitamin E, folate, thiamine, nonsodium minerals (magnesium, copper, potassium, and selenium), and other healthful bioactives (phytosterols and phenolic compounds) that confer nuts cardioprotective properties [57] and have been shown to improve diet quality in children [58].
• According to WHO recommendations, children and adolescents aged 2–15 y should not consume >2 g of salt/d to avoid undue increases in blood pressure (BP), whereas older adolescents (≥16 y of age) should not exceed 5 g of salt/d [59]. A good alternative to salt in the kitchen and at the table is to use herbs and spices, which improves flavor and adds color and taste to dishes.
• Traditional sauces prepared by simmering onions, garlic, and tomatoes with olive oil are known under different names around the Mediterranean Sea [e.g., *Sofrito* in Spain, *Soffritto* in Italy, *סופריטו* (*Sufritu*) in Israel, and *Τσιγάρισμα* (*Tsigarisma*) in Greece]. They are mainly used for dressing rice, pasta, and vegetable dishes and add flavor and bioactive compounds. This practice often serves as the starting point for several recipes, for example, Lathera recipes, typical in Greek cuisine [60,61].

#### Weekly

• Proteins are a key nutrient of any diet, present in essential foods that should be consumed to guarantee normal growth and development, such as legumes, lean and oily fish, eggs, poultry, and red meat. According to a joint FAO/WHO/United Nations University report, requirements for protein intake are around 0.9 g/kg/d for 3–18-y-old boys and 3–15-y-old girls [62]. Later, for 15–18-y-old girls, requirements decrease slightly to 0.8 g/kg/d [62]. Currently, animal protein intake exceeds recommendations in all age groups in Europe; therefore, decreasing animal protein intake is necessary, especially in young children.
• Eggs are an affordable and low-energy source of high-quality protein and key micronutrients such as nonsodium minerals, including calcium, and vitamins like vitamin D. The high cholesterol content of egg yolk has been a concern, but there is accumulating evidence that egg consumption does not increase cardiovascular disease risk [63]. In a large meta-analysis that included 39 prospective studies with nearly 2 million individuals, a 6% reduction in CVD incidence was reported with the consumption of ≤1 egg/d [64]. Hence, eggs can be consumed in amounts of ≤1 unit/d, but the recommendation continues to be the consumption of 3 servings per week.
• Pulses (legumes with exclusion of oleaginous seeds such as soybeans and peanuts) include chickpeas, lentils, white beans, fava beans, and lupins, among others. The recommendation is to consume ≥3 servings/wk because they are an important source of vegetable protein, complex carbohydrate, fiber, vitamins, nonsodium minerals, and bioactive phytochemicals, and their consumption reduces the potency of cardiometabolic risk factors and helps control body weight [65]. However, the effect of increased consumption of legumes on incidence of hard end points is still unclear, as meta-analyses of cohort studies have shown both a neutral association with incident CVD [66] and a beneficial association with moderate reduction of CVD events [67]. However, the overall evidence favors consumption of legumes to foster cardiometabolic health.
• Regarding fish, ≥3 servings/wk of fish or shellfish (seafood) are recommended. It is convenient to diversify the types of fish, both lean and oily, and preferably from sustainable fishing sources. Children aged 9 y or younger should avoid consuming species with potentially high mercury content, usually large predatory fish such as swordfish, bluefin tuna, and shark, whereas those aged 10–14 y should limit their consumption to 120 g/mo. A minimum of 2 servings/wk of seafood is necessary for kids to obtain key nutrients such as high-quality animal protein, vitamin D, calcium, iodine, selenium, and, foremost, long-chain n–3 (ω-3) PUFA, which are essential for neurocognition and retina development. Several observational studies suggest that maternal prenatal fish consumption is associated with better cognition and overall mental health in the offspring, although the results of clinical trials of fish oil supplementation with outcomes on cognition in children have been inconclusive. On the contrary, there is ample evidence of the beneficial effects of fish consumption on the incidence of coronary artery disease and stroke and total mortality risk [68].
• Consumption of red meat should be limited to a maximum of 2 servings/wk and processed meat such as cured meat, bacon, and sausages, to a maximum of 1 serving/wk. Adolescents tend to consume much more meat and meat products than recommended. A recent umbrella review of cohort studies assessing exposure to meat in the diet for NCD outcomes concludes that there is a consistent association of processed meat consumption with incidence of T2DM, but the evidence for an association with CVD is too weak for both unprocessed and processed meat [69]. On the contrary, substitution of poultry (white meat such as chicken and turkey) for red or processed meat is inversely related to cardiovascular disease risk, suggesting that white meat is a healthier alternative to red meat [70]. Nevertheless, data from substitution analyses suggest that replacing red and processed meat with beans, nuts, fish, poultry, or lean meat can reduce the incidence of several NCDs and premature death [71].
• A conventional recommendation concerning potatoes is that they should be limited due to their high GI, which would increase risk of glucose intolerance and T2DM. Additionally, potatoes have been associated with the reputedly unhealthy Western diet, wherein they are usually consumed fried, thus much increasing fat content, energy load, and, frequently, salt [72]. However, moving beyond the GI of potatoes, which is high when consumed as isolated foods but would decrease when they are part of mixed dishes with other vegetables or meat/fish, as customarily consumed in the MeDiet, and considering their rich nutrient composition, it can be argued that potatoes have had an undeservedly lost reputation as a healthy plant food. Potatoes are rich in starch and are also good sources of potassium, dietary fiber, and vitamins B and C. A study modeling replacement of 1 serving of potatoes for vegetables concluded that removing potatoes from childrens’ diets compromised the intake of potassium and other salutary nutrients [73]. A recent meta-analysis of cohort studies focused on risk of T2DM and gestational diabetes concluded that only consumption of potatoes higher than 100 g/d increase risk, whereas below this level they have a neutral effect or are even associated with reduced risk [74]. Taken together, the current evidence prompts a recommendation of eating ≤100 g of potatoes, 3 times/wk, preferably mixed with vegetables, as customary in the MeDiet.

#### Occasionally

• According to the WHO, free sugar intake for children should be reduced to a minimum of 10% of energy, a limitation that is advisable throughout the lifecourse, although a further reduction by 5% is associated with additional health benefits [75]. Most sweets, cakes and confectionery in general are made up of refined cereal flour, simple sugars, and unhealthy fats. Consequently, foods rich in simple sugars and SFAs and transfats are located at the top of the pyramid.
• Fresh fruit should be the usual dessert in the MeDiet, although sweets and cakes might be consumed occasionally [20].

### Foods outside the Mediterranean pyramid

Some foods are not strictly Mediterranean but are commonly consumed in Mediterranean countries since centuries. Some of these are healthful per se, whereas others are harmful and should be avoided.

• Chocolate. At present, 3 main types of chocolate are marketed: *1*) dark chocolate that contains a minimum of 70%–80% of cocoa solids and cocoa butter; *2*) milk chocolate, derived from high-fat milk and containing <20% of cocoa solids, sugar, and cocoa butter; and *3*) white chocolate, based on cocoa butter, sugar, and milk without cocoa solids. Most of the reported health benefits of cocoa are attributable to dark chocolate due to its high content in the flavanol class of polyphenols, which have been related to improved vascular function, lowered BP, improved insulin sensitivity, reduced risk of CVD and T2DM, and diminished platelet activation [76,77]. Given the evidence of the salutary effects of dark chocolate and the fact that, in spite of being an energy-dense food, its consumption within certain limits does not promote weight gain, chocolate can be consumed safely by children and adolescents.
• Ultraprocessed foods. Consumption of UPFs, in general, and foods and drinks rich in added sugars are quite high in children, contributing to a diet that is energy dense but nutrient poor. UPF consumption has been linked to obesity and increased cardiometabolic comorbidities in children and adolescents [78], while 1 subtype of UPFs, soft drinks or SSBs, which are widely available to them, are particularly harmful and clearly promote overweight and obesity [48], besides other health problems such as dental caries, hyperactivity, short sleep, elevated BP, and fatty liver [79], hence ideally their intake should be avoided. Sugar-rich solid foods are related to weight gain and increased adiposity, particularly abdominal obesity, and promote cardiometabolic risk factors in a similar way to sugary drinks [[80], [81], [82]].

### Environmental, sociocultural, and lifestyle components

The MeDiet purports to go beyond dietary recommendations and is meant to be a complete lifestyle portfolio, which encompasses individual, sociocultural, and environmental components, to preserve the cultural heritage and further overall health and well-being. It entails daily physical activity, including individual and collective games and sports, spending time with friends and family, sharing meals, and cooking time to exchange gastronomic culture, involving kids to help them establish healthy habits from childhood. Traditional culinary preparations made with seasonal, fresh, local foods, mainly of plant origin, with no UPFs or foods packed in plastic, as most UPFs are, make the MeDiet a coherent, environmentally sustainable dietary pattern.

### Physical activity

To keep an active, regular physical activity and a healthy mental status, it is critical to maintain good health and an appropriate energy balance, both during childhood and adulthood [83]. By the time the traditional MeDiet was described for the first time, it was associated with an active daily routine, in a context of heavy physical labor and absence of the effort-sparing technology currently available [20]. However, at present, physical inactivity is increasing in children, which represents an important health issue [32]. The reduced opportunities for physical activity that have resulted from urbanization, major changes in means of mechanic transportation, and the pervasiveness of leisure-time sedentary activities like TV watching, videogames, internet browsing, and smartphones are potential drivers of sedentariness. Thus, strategic interventions should be implemented to reverse this situation [84]. For kids aged 5–17 y, the WHO recommends engaging in ≥60 min daily of moderate-to-vigorous-intensity physical activity and emphasizes that a greater amount of physical activity will contribute to better health. Physical activity should be mainly aerobic, but vigorous-intensity exercise must be included a minimum of 3 times weekly. Around 80% of adolescents aged between 13 and 15 y, particularly girls, have been estimated to not meet these recommendations [85]. The MedLifestyle pyramid supports this advice (≥60 min/d of moderate-to-vigorous-intensity physical activity) and encourages children and adolescents to comply through collective and individual sports practice, focused on fun and respect, which has been associated with positive effects at an emotional and psychosocial level beyond those at the physical level. Active recreational games, where children can move, run, and jump outdoors or indoors, or at school during physical education classes, have been associated with better health indicators, self-esteem, and academic achievement [84].

### Sleep

A fundamental part of the MedLifestyle is sufficient, satisfactory, good quality sleep, which plays a crucial role in children’s and adolescents’ health and development [86]. The MedLifestyle pyramid encourages an adequate daily sleep routine, both at night and during the day through naps, adapted to individual needs, which are greatest in infants and decrease gradually until late adolescence. For children aged younger than 5 y, the WHO recommends sleeping between 10 and 13 h/d, including napping. On the contrary, scientific societies indicate that, for school-aged children (6–12 y), it is appropriate to sleep between 9 and 10–12 h/d and, in the case of adolescents (13–18 y) ∼8–10 h/night. Having regular sleep schedules, avoiding disturbing noises and screen devices, and minimizing caffeine or stimulant drink intake are important for good quality rest [87]. A recent large observational study conducted in 9–10-y-old children strongly suggests that insufficient sleep compromises neurodevelopment in early adolescence [88].

### Socialization

Spending time daily in socializing activities, within the family or with friends and classmates, is a relevant component of the Mediterranean way of living. Whenever possible, family members should eat together at the same table, sharing ≥1 meal/d (commensality). Another essential cultural component of the Mediterranean lifestyle, emphasized in the MeDiet pyramid for adults [24], is the pleasure, friendliness, and happiness derived from enjoying meals together (conviviality). Both commensality and conviviality can translate into manifold health benefits, such as improved mood, reduced stress, better nutrient intake, and improved general well-being [89]. It is important to involve kids in food selection, meal planning and preparation, allowing them to create strong bonds between different generation of family members, exchange gastronomic and cultural traditions, and develop complementary culinary and communication skills. This allows building healthy habits from the earliest age [90] and a sense of community [91], while enabling young children to incorporate mealtimes as a routine around the table, to learn manners and to realize that this may be a pleasant and relaxing moment where one can share, talk, and go over the day’s events. It is necessary to recall that, at this point, adults can be positive role models for children. Furthermore, school mealtimes [92], extracurricular activities, birthdays, and other celebrations are situations where children and adolescents can interact with adults and develop social skills.

### Traditional recipes and preparations

The MeDiet is rich in traditional recipes and preparations, which have been transmitted from generation to generation. The MeDiet abounds in diverse plant foods containing a variety of bioactive compounds that have been associated with positive health effects, and culinary preparations based on EVOO can enhance both flavor and antioxidant power, which is associated with cardiometabolic benefit and improved cognition [93,94]. Slow cooking techniques, like stews, where most of the nutritional value of food is preserved, are typically used [33,[95], [96], [97]]. A fitting example of Mediterranean culinary preparations are the aforementioned recipes of sauces made by simmering EVOO, garlic, onion, and tomatoes. Although it is the base of many dishes in the MeDiet, which enhances the nutritional qualities of these ingredients, this preparation has been associated with beneficial cardiovascular, anti-inflammatory, and anticancer effects [60,61].

### Sustainable food pattern: fresh, local, and seasonal foods

Finally, sustainability, a key feature of the MeDiet, needs to be highlighted [15,[98], [99], [100], [101], [102], [103]]. Currently, many of the foods commercialized in Western countries are associated with a high environmental impact, making the present consumption pattern unsustainable in the long-term [104,105]. Therefore, it is imperative to modify consumer habits to solve this situation. MeDiet recipes include a wide variety of fresh, local, and seasonal foods, so that diet changes during the year adapt to the natural seasonality of each crop [29]. Thus, winter MeDiet recipes are rich in cabbage, turnips, broccoli, and pumpkins with oranges, mandarins, and other citrus fruits for dessert, whereas summer recipes are full of garden products like peppers, eggplants, tomatoes, green beans, lettuce, and cucumbers, as well as seasonal fruits like melon, watermelon, peaches, or cherries. The low-to-moderate presence in the MeDiet of animal-based foods, like meat or dairy products in general and red and processed meat in particular, is a key sustainability factor. Concerning UPFs, not only they are nutrient poor, but also their tendency to be packed or covered in plastic is antagonistic to the MeDiet. The use of local crop varieties and native breeds enhance the Mediterranean cultural heritage and the adaptation of agriculture to climate change through diet.

Changing the dietary pattern toward a plant-based diet such as the MeDiet can significantly reduce greenhouse gas emissions, while promoting biodiversity, conservation, and sustainable natural resources management because the MeDiet is associated with a lesser footprint on water, soil, and energy resources [[105], [106], [107]].

Local, seasonal products reduce the environmental impact attributable to food transportation, distribution, and storage [29,107]. Simultaneously, associated agricultural costs are reduced, and the local economy is empowered. Moreover, fresher foods better maintain their nutritive and sensory qualities and, in turn, help preserve the biodiversity of plant and animal species.

### Conclusions

In the era of personalized nutrition, dietary recommendations should be adapted to different stages of life, including children (older than 3 y) and adolescents. We present an updated version of the Mediterranean Lifestyle Pyramid addressed to kids, as well as health professionals, teachers, and stakeholders. Fruit, vegetables, legumes, nuts, wholegrains, and EVOO continue to be at the basis of the pyramid, but the importance of an adequate intake of fish, dairy products, and meat during these particular ages, when body and brain development occurs, is also considered. The promotion of physical activity, adequate sleep, and good emotional health are emphasized, as well as the consumption of seasonal and local products and overall sustainability.

This new pyramid is expected to contribute to improving adherence to the Mediterranean dietary pattern and lifestyle in children and adolescents, while emphasizing the promotion of seasonal and local products and overall sustainability. Improving dietary habits in early stages of life should increase health in adulthood and reduce future incidence of noncommunicable chronic diseases.

The MeDiet and its graphic representation in the MedLifestyle pyramid is, therefore, a health-promoting tool, not only for adults and children but also for the entire planet because it promotes the diversity of the species, respect for the earth, and the local economy. This pyramid should be spread to families, school canteens, dining halls, and restaurants with the help of governments and scientific institutions.

## Author contributions

The authors’ responsibilities were as follows – all authors: contributed to the study concept and design and to the acquisition, analysis, or interpretation of data; RE, RC, JA, ER: wrote the first draft of the manuscript; all authors: critically revised the manuscript for important intellectual content, approved the final version, and had final responsibility for the decision to submit for publication. The Mediterranean diet Foundation organized the XII Congress on the Mediterranean diet, held in Barcelona, which bought together most of the international experts contributing to this manuscript and initiated a forum to develop these guidelines.

## Declaration of Generative AI and AI-assisted technologies in the writing process

During the preparation of this work the author(s) did use ChatGPT for grammar assistance.

## Funding

The authors reported no funding received for this study.

## Conflict of interest

RE reports administrative support by Fundacion Dieta Mediterranea, Barcelona (Spain); grants from Carlos III Health Institute, European Comission (Brussells, Belgium), National Institute of Health (Bethesda, MD), Grand-Fountain Laboratories (Spain), and Fundacion de Investigación sobre Vino y Nutricion (Vilafranca, Spain); consulting or advisory for Cerveza y Salud (Madrid, Spain) and Dallant Laboratories (Spain); speaking and lecture fees from Wine and Culinary International Forum (Barcelona, Spain); travel reimbursement from Karolinska Institute, Menarini Laboratories (Sweden), Iberoamerican Foundation for Nutrition, Italian Pavilion, EXPO DUBAI 2020, Vatican City, and Fundacion de Investigación sobre Vino y Nutricion (Vilafranca, Spain). AMR-L reports paid expert testimony from Fundacion Dieta Mediterranean, Barcelona, Spain. SC-B reports is an employee of Alfonso Martín Escudero Foundation. JF reports grants from Spanish Ministry of Science, Innovation, and Universities. RML-R reports grants from Carlos III Health Institute, European Comission (Brussells, Belgium), National Institute of Health (Bethesda, MD), a Cerveza y Salud (Madrid, Spain); nonfinancial support from Ecoveritas SA (Spain); consulting or advisory for UNIDECO SA; and travel reimbursement for Wine in Moderation (Brussels) and ADVENTIA SA (Spain). ER reports speaking and lecture fees from Alexion Pharmaceuticals and Spanish Arteriosclerotic Society and travel reimbursement from Alexion Pharmaceuticals, California Walnuts, and International Nut and Dried Fruit Council. AV reports travel reimbursement from Italian Society of Human Nutrition. The other authors report no conflicts of interest.
