# Supplementary material for: A New Mediterranean Lifestyle Pyramid for Children and Youth: A Critical Lifestyle Tool for Preventing Obesity and Associated Cardiometabolic Diseases in a Sustainable Context
Source: Adv Nutr. 2025 Jan 21;16(3):100381. doi: 10.1016/j.advnut.2025.100381 (PMC11875175; doi:10.1016/j.advnut.2025.100381)
Supplement: Multimedia component 1 [file mmc1.docx]

**Supplemental Material**

Although the Mediterranean diet does not fit into a 'single plate' due to the importance of the timing and composition of the main meals, breakfast, lunch, and dinner in this diet do not conform to a single plate either. However, considering the popularity of the Harvard plate, three designed plates—one for breakfast, one for lunch, and one for dinner—are included to help better understand the Mediterranean diet.

**Figure 1S**

A graphic representation of three dishes corresponding to the three main meals: breakfast, lunch, and dinner.

.
